# DNA recovery after sequential processing of latent fingerprints on copy paper

**DOI:** 10.1111/1556-4029.14881

**Published:** 2021-09-09

**Authors:** Abigail S. Bathrick, Sarah Norsworthy, Dane T. Plaza, Mallory N. McCormick, Donia Slack, Robert S. Ramotowski

**Affiliations:** ^1^ Bode Technology Lorton VA USA; ^2^ Forensic Technology Center of Excellence RTI International Research Triangle Park NC USA; ^3^ Forensic Services Division United States Secret Service Washington DC USA; ^4^ Present address: National Institute of Standards and Technology Gaithersburg MD USA

**Keywords:** DNA profiling, fingermarks, fingerprint development, forensic analysis, latent fingerprints, touch DNA

## Abstract

Forensic examiners must determine whether both latent fingerprint development and DNA profiling can be performed on the same area of an evidence item and, if only one is possible, which examination offers the best chance for identification. Latent fingerprints can be enhanced by targeting different components of fingerprint residues with sequential chemical treatments. This study investigated the effects of single‐reagent and sequential latent fingerprint development processes on downstream DNA analysis to determine the point at which latent fingerprint development should be stopped to allow for DNA recovery. Latent fingerprints deposited on copy paper by one donor were developed using three sequential processes: 1,8‐diazafluoren‐9‐one (DFO) → ninhydrin → physical developer (PD); 1,2‐indanedione‐zinc (IND‐Zn) → ninhydrin → PD; and IND‐Zn → ninhydrin → Oil Red O (ORO) → PD. Samples were examined after the addition of each chemical treatment. DNA was collected with cotton swabs, extracted, quantified, and amplified. DNA yields, peak heights, number of alleles obtained, and percentage of DNA profiles eligible for CODIS upload were examined. DNA profiles were obtained with varying degrees of success, depending on the number and type of treatments used for latent fingerprint development. The treatments that were found to be the least harmful to downstream DNA analysis were IND‐Zn and IND‐Zn/laser, and the most detrimental treatments were DFO, DFO/laser, and PD. In general, as the number of treatments increase, the opportunities for DNA loss or damage also increase, and it is preferable to use fewer treatments when developing latent fingerprints prior to downstream DNA processing.

**Highlights**

- The effects of latent fingerprint development processes on subsequent DNA analysis were investigated.
- Development processes included DFO, DFO/laser, IND‐Zn, IND‐Zn/laser, ninhydrin, ORO, and PD.
- Sequential processing may be considered depending on the number and type of treatments used.
- Results demonstrated that fewer treatments are preferable.
- DFO and PD are not recommended when performing downstream DNA analysis.

## 1 INTRODUCTION

Disciplines within the forensic field must often work together to ensure the most comprehensive examination of evidence is accomplished. In today's crime laboratories, it is not uncommon for fingerprint examiners and DNA analysts to visually examine evidence to determine the best course of action prior to processing and to develop a joint plan of action for downstream forensic testing of the item of interest. Because latent fingerprint and forensic DNA testing can be considered destructive processes, the questions often asked are whether or not both examinations can be performed on the same area and, if only one is possible, which is likely to offer the best chance for identification. Previous studies have shown that partial to full DNA profiles can still be obtained from a single fingerprint on paper substrates after latent print processing [1]. Various groups have even demonstrated that while the quality of DNA profiles may be negatively affected, quantifiable DNA can be obtained from fingerprints treated with single development reagents such as ninhydrin, 1,8‐diazafluoren‐9‐one (DFO), and 1,2‐indanedione‐zinc (IND‐Zn) [1, 2, 3, 4, 5].

Latent fingerprints are primarily comprised of eccrine sweat released from pores between the friction ridges, sebaceous secretions from other areas of the body, and contaminants from the environment thus producing an impression of the ridges when deposited [6]. Eccrine sweat is comprised of a complex variety of compounds [7], including water (20%–70%) [8, 9] and quantifiable amounts of amino acids that react with reagents, such as DFO [10], ninhydrin [11], and IND‐Zn [12], through distinct mechanisms. Additionally, fingerprint residues contain sebaceous oils that are transferred to the fingers when an individual touches sebaceous gland‐rich areas such as the face and scalp [6]. When deposited on porous surfaces, the lipids found in these sebaceous oils can be detected by chemicals such as physical developer (PD) or Oil Red O (ORO). Because each of these development processes reacts with different components found in fingerprint residue, it has been determined that the development of latent fingerprints can be enhanced by a targeted sequential processing scheme whereby different attributes of fingerprint residues are developed in a stepwise manner [13, 14]. While various studies have been conducted to determine the downstream effects of single‐process latent fingerprint development on DNA analysis [1, 2, 3, 4, 5, 15, 16], the extent to which routine sequential latent fingerprint development processes affect the recovery of DNA is unknown.

The aim of this study was to evaluate the effects of single‐reagent and sequential latent fingerprint development processes on downstream DNA analysis. If an effect was determined, the secondary goal was to determine at which step in the process the DNA was affected. Three sequential processes for developing latent fingerprints on paper were evaluated: (1) DFO (visualized with a 532 nm laser), followed by ninhydrin, followed by PD [14, 17, 18, 19]; (2) IND‐Zn (visualized with a 532 nm laser), followed by ninhydrin, followed by PD [14, 20]; and (3) IND‐Zn, followed by ninhydrin, followed by ORO, followed by PD [13, 17]. DFO was popularized as a component of single‐reagent and sequential processes in the 1990's [21] and became the second most widely used reagent for developing latent fingerprints on porous surfaces by 2004 [22]. IND‐Zn has been identified as the successor to DFO [19, 20, 23, 24, 25]; however, DFO is still used by some practitioners. DFO [26] and IND‐Zn [20] may both be followed with ninhydrin to develop additional ridge details. Further treatment with reagents like PD and ORO can sometimes develop additional latent prints not previously visualized [13].

## 2 MATERIALS AND METHODS

### 2.1 Solution preparation

The materials and methods for preparing the chemical solutions used for latent fingerprint development can be found in Table 1. Reverse osmosis‐deionized (RO‐DI) water was generated using an Elga Purelab Option‐S 7/15 (~18 MΩ).

**TABLE 1:** Preparation of chemical solutions used for latent fingerprint development

Solution	Component
DFO	500 mg DFO (Lumichem, ODV, Inc.)
	100 ml Methanol (Peroxide‐Free/Sequencing, Fisher Scientific)
	100 ml ethyl acetate (ACS grade, Fisher Scientific)
	20 ml acetic acid (ACS grade, Spectrum)
	780 ml petroleum ether (ACS grade, Fisher Scientific)
IND‐Zn	0.8 g 1,2‐indanedione (Casali Institute of Applied Chemistry)
	90 ml ethyl acetate (ACS grade, Fisher Scientific)
	10 ml acetic acid (ACS grade, Spectrum)
	80 ml zinc chloride solution
	820 ml petroleum ether (ACS Grade, Fisher Scientific)
Zinc chloride	0.4 g zinc chloride (ACS grade, Millipore Sigma)
	10 ml absolute ethanol (ACS grade, Spectrum)
	1 ml ethyl acetate (ACS grade, Fisher Scientific)
	190 ml petroleum ether (ACS grade)
Ninhydrin	6 g ninhydrin (Sirchie)
	50 ml absolute ethanol (ACS grade, Spectrum)
	950 ml petroleum ether (ACS grade, Fisher Scientific)
ORO	1.54 g ORO (Millipore Sigma)
	770 ml methanol (Peroxide‐Free/Sequencing, Fisher Scientific)
Sodium hydroxide	9.2 g sodium hydroxide (ACS grade, Fisher Scientific)
	230 ml RO‐DI water
PD working	900 ml REDOX stock solution
	40 ml detergent stock solution
	50 ml silver nitrate stock solution
REDOX stock	900 ml RO‐DI water
	30 g ferric nitrate nonahydrate (ACS grade, Fisher Scientific)
	80 g ferrous ammonium sulfate hexahydrate (ACS grade, Fisher Scientific)
	20 g citric acid monohydrate (USP grade, Spectrum)
Detergent stock	1 L RO‐DI water
	3 g n‐dodecylamine acetate (Pfalz & Bauer)
	3 ml Tween 20 (Millipore Sigma)
Silver nitrate stock	10 g silver nitrate (ACS grade, Fisher Scientific)
	50 ml RO‐DI water
Malic acid	25 g Malic acid (Spectrum)
	1000 ml RO‐DI water

### 2.2 Fingerprint preparation

A total of 144 fingerprints were prepared on standard‐weight copy paper (Staples) by one donor over the course of 9 days. Fingerprints were obtained with informed consent. A single donor was used to mitigate DNA yield variability associated with different individuals' propensities to shed epithelial cells [27]. Paper was cut into 1.5″ × 2.0″ sections and was decontaminated by short‐wave UV irradiation in a Spectronics Spectrolinker™ XL‐1500 with a 254 nm bulb. Substrates were placed on a desktop scale, and the index, middle, and ring fingers from both hands were used to deposit fingerprints by pressing down until 2 kg was reached after a total substrate contact time of approximately 1 s. Next, the finger was removed from the substrate. To mimic real‐world evidentiary‐handled documents, fingerprints were not charged with additional sebaceous oils, handwashing was kept to a minimum, and at least 2 h elapsed after handwashing and between fingerprint depositions.

### 2.3 Fingerprint processing

Latent fingerprints were treated with single‐reagent or sequential latent fingerprint development processes (Table 2). Eight replicates were processed with either DFO, ninhydrin, ORO, or PD, and 10 replicates were processed with either DFO/laser, IND‐Zn, or IND‐Zn/laser. Three sequential processes were examined: (1) DFO/laser → ninhydrin → PD; (2) IND‐Zn/laser → ninhydrin → PD; and (3) IND‐Zn → ninhydrin → ORO → PD. Within each process, 10 replicates were examined after the addition of each treatment to determine its effect on DNA recovery. For example, for IND‐Zn → ninhydrin → ORO → PD, a total of 30 replicates were processed. Ten replicates were processed with IND‐Zn + ninhydrin, 10 replicates were processed with IND‐Zn + ninhydrin + ORO, and 10 replicates were processed with IND‐Zn + ninhydrin + ORO + PD. Additionally, 12 untreated fingerprints were processed as controls for comparison to the treated fingerprints.

**TABLE 2:** The sample size (*n*) for each processing type and treatment

Processing type	Treatment	Sample size (*n*)
n/a	Untreated	12
Single	DFO	8
Reagent	DFO/Laser	10
	IND‐Zn	10
	IND‐Zn/Laser	10
	Ninhydrin	8
	ORO	8
	PD	8
Sequential	DFO/Laser + Ninhydrin	10
	DFO/Laser + Ninhydrin + PD	10
	IND‐Zn/Laser + Ninhydrin	10
	IND‐Zn/Laser + Ninhydrin + PD	10
	IND‐Zn + Ninhydrin	10
	IND‐Zn + Ninhydrin + ORO	10
	IND‐Zn + Ninhydrin + ORO + PD	10

#### 1,8‐Diazafluoren‐9‐One

DFO was prepared following the Federal Bureau of Investigation's (FBI) guidelines for developing latent fingerprints [18]. Samples were briefly soaked in the reagent, air dried, and placed in an Isotemp^®^ model 106G oven (Thermo Fisher Scientific) for 20 min at 100°C for development.

#### 1,2‐Indanedione‐Zinc

IND‐Zn was prepared following Ramotowski [28]. Samples were briefly soaked in the reagent, air dried, and placed in an environmental chamber (Caron) for 15 min at 80°C and 65% relative humidity for development.

#### DFO/Laser & IND‐Zn/Laser

DFO and IND‐Zn are typically used in conjunction with an alternate light source (ALS) to visualize the developed fingerprints [29]. To determine if the ALS exposure negatively impacts DNA recovery, fingerprints treated with DFO and IND‐Zn were collected for subsequent DNA analysis with and without exposure to a 532 nm light for approximately 10 s using a 4‐watt TracER™ laser (Coherent, Inc.).

#### Ninhydrin

Ninhydrin was prepared following Ramotowski [28]. Samples were briefly soaked in the reagent, air dried, and placed in an environmental chamber (Caron) for 15 min at 80°C and 65% relative humidity for development.

#### Oil Red O

Oil Red O was prepared by combining ORO and sodium hydroxide solutions following guidelines from Beaudoin [30]. Samples were immersed in the ORO solution for approximately 60 min while being agitated on an orbital shaker (Barnstead International), rinsed in tap water for approximately 10 min while being agitated, and air‐dried overnight.

#### Physical developer

Physical developer working solution was prepared following Houlgrave et al. [31] Samples were immersed in an RO‐DI water bath for 10 min while being agitated on an orbital shaker (Barnstead International). The water was drained and replaced with a malic acid solution. Samples were immersed in the acid for 10 min while being agitated. The acid was drained and replaced with the PD working solution. Samples were immersed in the PD solution 20 min while being agitated. The PD solution was drained and replaced with tap water. Samples were immersed in tap water for 10 min while being agitated and were air‐dried overnight.

### 2.4 Sample collection

DNA was collected directly from the paper with 100% cotton Bode SecurSwabs (Bode Technology) by wetting the tip of the swab with 1–2 drops sterile DNA grade water from a 3 ml AddiPak brand water vial, swabbing the area where the fingerprint was deposited and/or visualized, turning the swab to the dry portion of the swab head, and swabbing the area again. To determine where in the sequential process the DNA may be affected, DNA was collected after each treatment was added.

### 2.5 DNA analysis

Samples were extracted using the Qiagen^®^ EZ1^®^ DNA Investigator Kit (Qiagen). Lysis was performed by incubating the whole swab head in 95 μl buffer G2, 95 μl ddH_2_O, and 20 μl Proteinase K at 56°C with shaking at 900 rpm for 1 h. To collect the lysates, samples were transferred to spin baskets and centrifuged at 20K × *g* for 2 min. The spin basket containing the swab head was discarded, and 1 μl carrier RNA (1 mg/μl) was added to the collected lysate. The samples were purified on an EZ1 Advanced Instrument (Qiagen) using the trace protocol and eluting into 50 μl TE buffer. Next, 2 μl of each DNA extract were quantified in 11 μl reaction volumes using the Quantifiler^®^ Trio DNA Quantification Kit (Thermo Fisher) on an ABI™ 7500 Real‐Time PCR System with HID Real‐Time PCR Software (Thermo Fisher). Samples containing less than 0.1 ng/μl DNA were concentrated to 10 ul using Microcon^®^ DNA Fast Flow centrifugal filter units (EMD Millipore) treated with 1 µl carrier RNA (1 mg/µl) in TE buffer. Samples were amplified for 28 cycles on a GeneAmp™ PCR System 9700 (Thermo Fisher) using a GlobalFiler^®^ PCR Amplification Kit (Thermo Fisher) in 12.5 μl reaction volumes with a target template DNA concentration of 1 ng for each amplification reaction. Samples containing more than 1 ng DNA were diluted to 0.5 ng/μl, and 2 μl were amplified. Samples containing less than 1 ng DNA were amplified at the maximum DNA input volume. Capillary electrophoresis (CE) was performed on a 3500xL Genetic Analyzer (Thermo Fisher) using 1 µl amplification product, POP‐4™ polymer (Thermo Fisher), and 3500/3500 xL Data Collection Software, v3.1. The injection time was 24 s with an injection voltage of 1.2 kV. Results were analyzed using ABI GeneMapper^®^ ID‐X v1.4 software (Thermo Fisher) with an analytical threshold (AT) of 125 relative fluorescent units (RFU) and a stochastic threshold (ST) of 600 RFU. Reagent blanks, substrate negative controls, extraction positives, positive amplification controls, and negative amplification controls were also processed.

### 2.6 Data analysis

The effects of the single‐reagent and sequential latent fingerprint development treatments were evaluated by examining DNA yield, peak height values, number of alleles obtained, and percentage of profiles eligible for Combined DNA Index System (CODIS) upload (percentage of profiles containing alleles from at least eight loci with a match rarity of at least one in ten million [32]). For each sample, the obtained profile was compared with the known genotype of the donor and expected alleles that were not present were assigned peak heights of 0 RFU. The maximum number of alleles that could be obtained was 46. Homozygous genotypes were expected at D13S317 and D19S433 and were counted as two alleles when the peak heights were above the ST, in which case peak heights were divided in half and applied to each allele.

JMP^®^ Software (SAS Institute Inc.) was used to perform statistical analysis of the data. The Shapiro‐Wilk test for normality was performed to determine whether the data were normally distributed. For DNA yield, number of alleles obtained, and peak height, pairwise comparisons of treatments were performed using Steel–Dwass all pairs, a nonparametric analog to Tukey's honest significant difference test. The Wilcoxon test (i.e., Mann–Whitney test) was used to compare peak heights for lower (<200 bp) and higher (>200 bp) molecular weight loci. For all statistical analyses, differences were considered statistically significant at *p* < 0.05.

## 3 RESULTS

### 3.1 Latent fingerprints developed using single‐reagent processes

**FIGURE 1:**
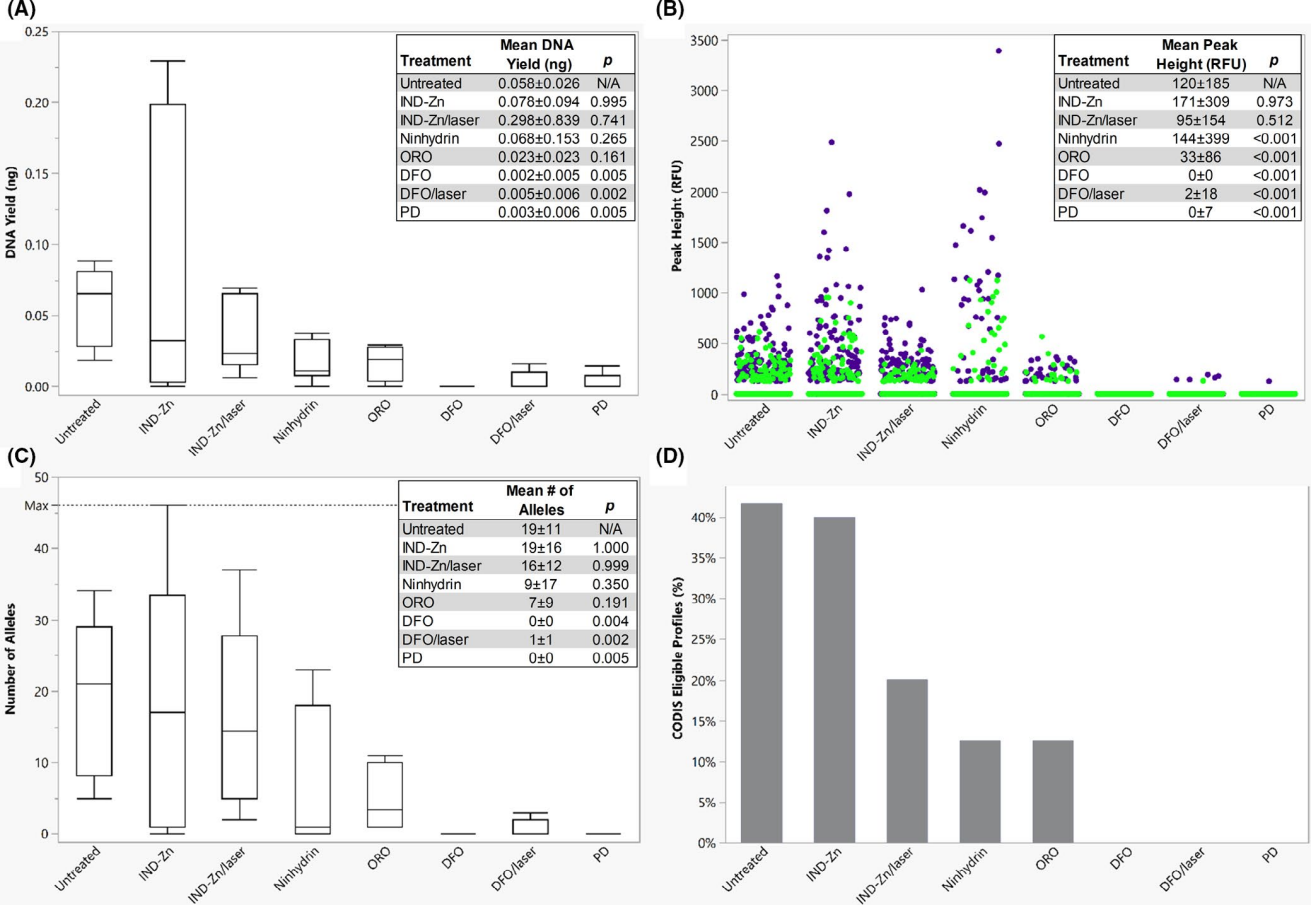
Latent fingerprints developed using single‐reagent processes. (A) A boxplot of the DNA yield for each process, showing the median and the 1st and 3rd quartiles. The whiskers represent 1.5 times the interquartile range. (B) The distribution of peak heights for lower (<200 bp, blue dot) and higher (>200 bp, green dot) molecular weight loci, with an AT of 125 RFU. (C) A boxplot of the number of alleles obtained following each process, showing the median and the 1st and 3rd quartiles. The whiskers represent 1.5 times the interquartile range. (D) The percentage of profiles eligible for CODIS upload (≥8 loci with a match rarity of at least 1 in 10 million) for each process. All overlaid boxes indicate means ± standard deviations and *p*‐values obtained from comparisons to the untreated samples (Steel‐Dwass All Pairs, *α* = 0.05) [Color figure can be viewed at wileyonlinelibrary.com]

DNA yields, peak heights, number of alleles obtained, and percentage of profiles eligible for CODIS upload obtained from the untreated latent fingerprints and the latent fingerprints treated with either DFO, DFO/laser, IND‐Zn, IND‐Zn/laser, ninhydrin, ORO, or PD were compared (Figure 1). The DNA yields of the untreated samples ranged from 0.018 to 0.089 ng with a median of 0.066 ng and were significantly higher than the yields for samples treated with DFO (*p* = 0.005), DFO/laser (*p* = 0.002), and PD (*p* = 0.005). Samples treated with IND‐Zn also produced significantly higher DNA yields than DFO (*p* = 0.010), DFO/laser (*p* = 0.015), and PD (*p* = 0.021). DNA quantity is not necessarily indicative of the DNA quality, and DNA concentration alone cannot be used to assess the effects of the treatments on the DNA. Fingerprints treated with IND‐Zn and IND‐Zn/laser generated peak height values that were not significantly different than the untreated samples, and all other treatments demonstrated significantly lower peak height values than the untreated samples (*p* < 0.001). Additionally, the untreated samples exhibited significantly greater peak heights for lower molecular weight loci (<200 bp) than higher molecular weight loci (>200 bp; *p* < 0.001). This significant difference can be attributed to the alleles that dropped out, which were assigned peak heights of 0 RFU. When these alleles were excluded from analysis, the difference between the peak heights at lower and higher molecular weight loci was no longer significant (*p* = 0.084). Stochastic effects and allelic dropout are commonly observed at higher molecular weight loci in low‐yield DNA samples. Statistically significant differences between the lower and higher molecular weight loci were also observed for samples processed with IND‐Zn (*p* < 0.001) and IND‐Zn/laser (*p* < 0.001), although the difference observed for IND‐Zn was no longer significant after the alleles with 0 RFU peak heights were excluded from analysis (*p* = 0.163). The DFO, DFO/laser, and PD samples exhibited high degrees of allelic dropout, and no samples treated with DFO, 30% of samples treated with DFO/laser, and 12.5% of samples treated with PD produced peaks above the AT. Compared with the untreated samples, samples treated with the following reagents generated significantly fewer alleles: DFO (*p* = 0.004), DFO/laser (*p* = 0.002), and PD (*p* = 0.005). Only two samples produced full profiles, one treated with IND‐Zn and one treated with ninhydrin. Across all samples treated with single‐reagent processes, 13% of profiles met the threshold for CODIS eligibility. For untreated samples, 42% of profiles were CODIS eligible.

To determine if laser exposure negatively impacted DNA recovery, latent fingerprints treated with DFO and IND‐Zn were collected for subsequent DNA analysis with and without exposure to the laser. This variation in processing did not lead to statistically significant differences in DNA yield, number of alleles obtained, or peak height for either chemical treatment.

### 3.2 Sequentially treated latent fingerprints, DFO/Laser → Ninhydrin → PD

**FIGURE 2:**
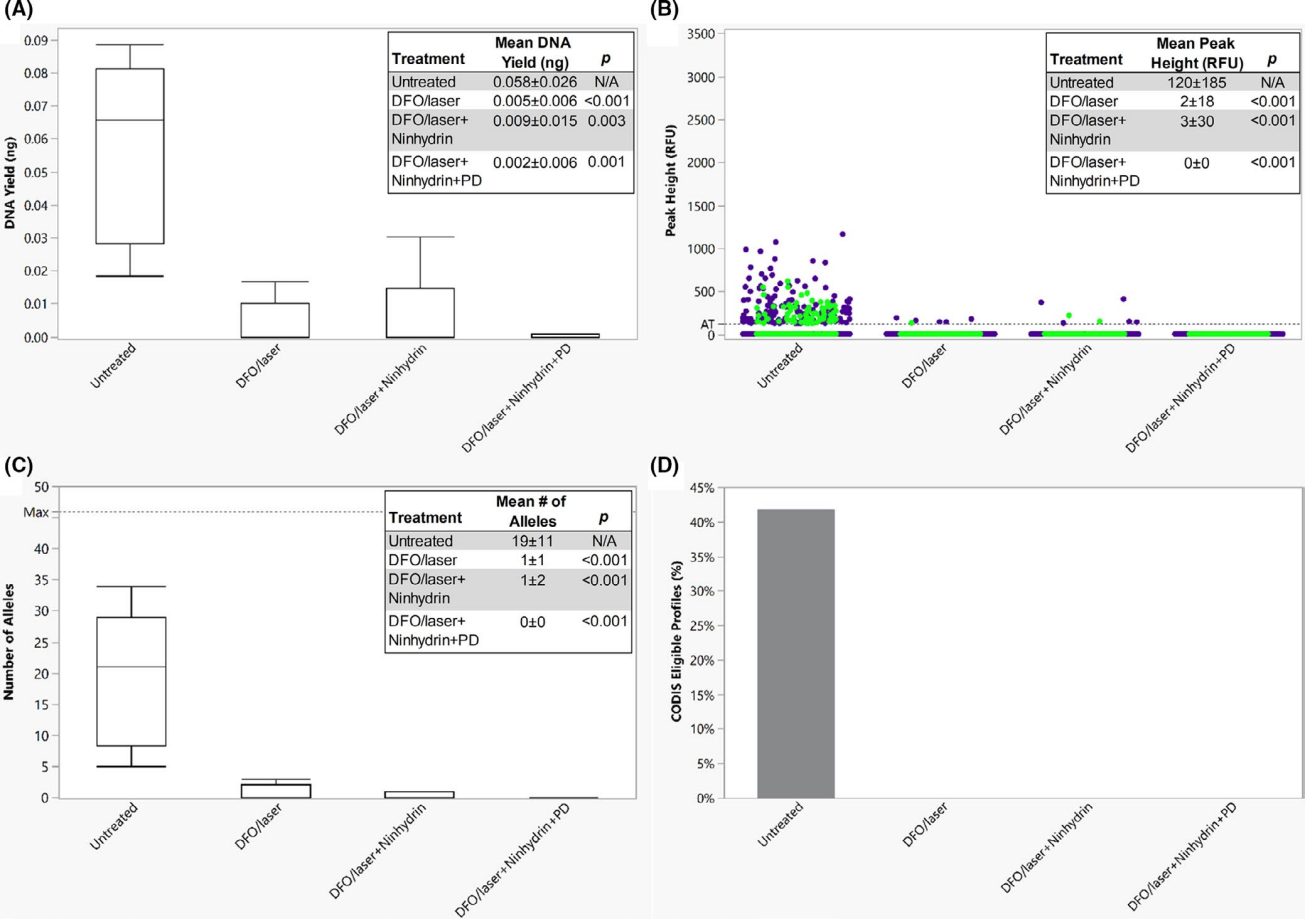
Latent fingerprints developed using sequential processes. (A) A boxplot of the DNA yield for each process, showing the median and the 1st and 3rd quartiles. The whiskers represent 1.5 times the interquartile range. (B) The distribution of peak heights for lower (<200 bp, blue dot) and higher (>200 bp, green dot) molecular weight loci, with an AT of 125 RFU. (C) A boxplot of the number of alleles obtained following each process, showing the median and the 1st and 3rd quartiles. The whiskers represent 1.5 times the interquartile range. (D) The percentage of profiles eligible for CODIS upload (≥8 loci with a match rarity of at least 1 in 10 million) for each process. All overlaid boxes indicate means ± standard deviations and *p*‐values obtained from comparisons to the untreated samples (Steel‐Dwass All Pairs, *α* = 0.05) [Color figure can be viewed at wileyonlinelibrary.com]

Latent fingerprints treated sequentially with DFO/laser, ninhydrin, and PD were compared with the untreated latent fingerprints using the aforementioned metrics (Figure 2). All sequentially treated samples demonstrated statistically significant decreases in DNA yield (DFO/laser and DFO/laser + ninhydrin + PD, *p* < 0.001; DFO/laser + ninhydrin, *p* = 0.003), peak height (*p* < 0.001), and number of alleles (*p* < 0.001) compared with the untreated samples. While untreated samples obtained 19 ± 11 alleles, samples treated with DFO/laser and with DFO/laser + ninhydrin obtained 1 ± 1 and 1 ± 2 alleles, respectively. Samples treated with DFO/laser + ninhydrin + PD produced no peaks above the AT. No treated samples produced profiles that met the threshold for CODIS eligibility.

### 3.3 Sequentially treated latent fingerprints, IND‐Zn/Laser → Ninhydrin → PD

**FIGURE 3:**
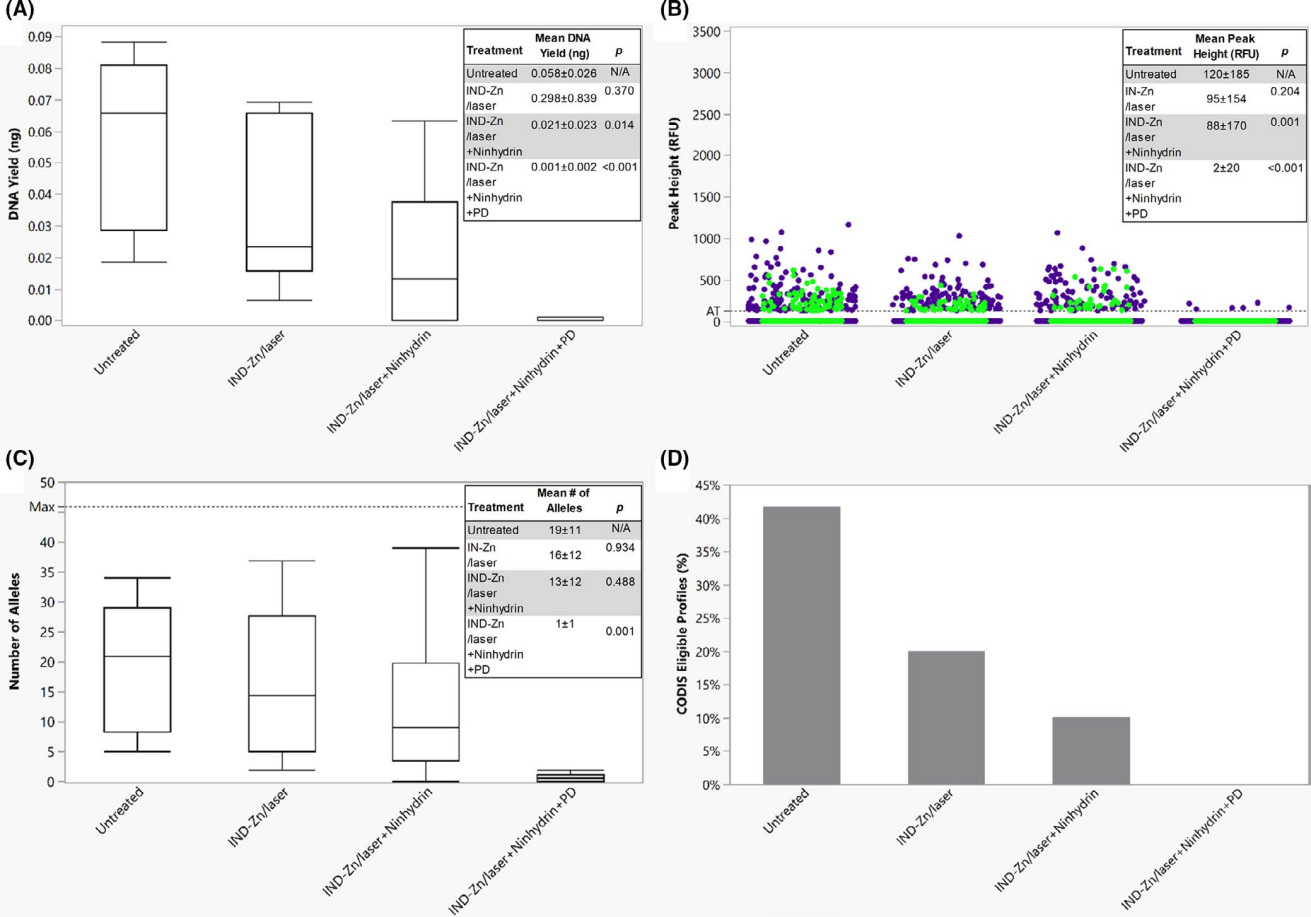
Latent fingerprints developed using sequential processes. (A) A boxplot of the DNA yield for each process, showing the median and the 1st and 3rd quartiles. The whiskers represent 1.5 times the interquartile range. (B) The distribution of peak heights for lower (<200 bp, blue dot) and higher (>200 bp, green dot) molecular weight loci, with an AT of 125 RFU. (C) A boxplot of the number of alleles obtained following each process, showing the median and the 1st and 3rd quartiles. The whiskers represent 1.5 times the interquartile range. (D) The percentage of profiles eligible for CODIS upload (≥8 loci with a match rarity of at least 1 in 10 million) for each process. All overlaid boxes indicate means ± standard deviations and *p*‐values obtained from comparisons to the untreated samples (Steel‐Dwass All Pairs, *α* = 0.05) [Color figure can be viewed at wileyonlinelibrary.com]

Latent fingerprints treated sequentially with IND‐Zn/laser, ninhydrin, and PD and untreated latent fingerprints were also compared using the previously stated metrics (Figure 3). Significantly lower DNA yields were obtained for samples treated with IND‐Zn/laser + ninhydrin (*p* = 0.014) and IND‐Zn/laser + ninhydrin + PD (*p* < 0.001) when compared with the untreated samples. Latent fingerprints treated with IND‐Zn/laser + ninhydrin and IND‐Zn/laser + ninhydrin + PD also exhibited statistically significant decreases in peak height when compared with the untreated samples (*p* < 0.001). The mean number of alleles obtained decreased after each treatment, and significantly fewer alleles were obtained from IND‐Zn/laser + ninhydrin + PD when compared with the untreated samples (*p* = 0.001), IND‐Zn/laser‐treated samples (*p* = 0.002), and IND‐Zn/laser + ninhydrin‐treated samples (*p* = 0.009). With the addition of each treatment, the percentage of profiles eligible for CODIS upload also decreased. Respectively, 42%, 20%, 10%, and 0% of the untreated, IND‐Zn/laser, IND‐Zn/laser + ninhydrin, and IND‐Zn/laser + ninhydrin + PD samples met the threshold for CODIS eligibility.

### 3.4 Sequentially treated latent fingerprints, IND‐Zn → Ninhydrin → ORO → PD

**FIGURE 4:**
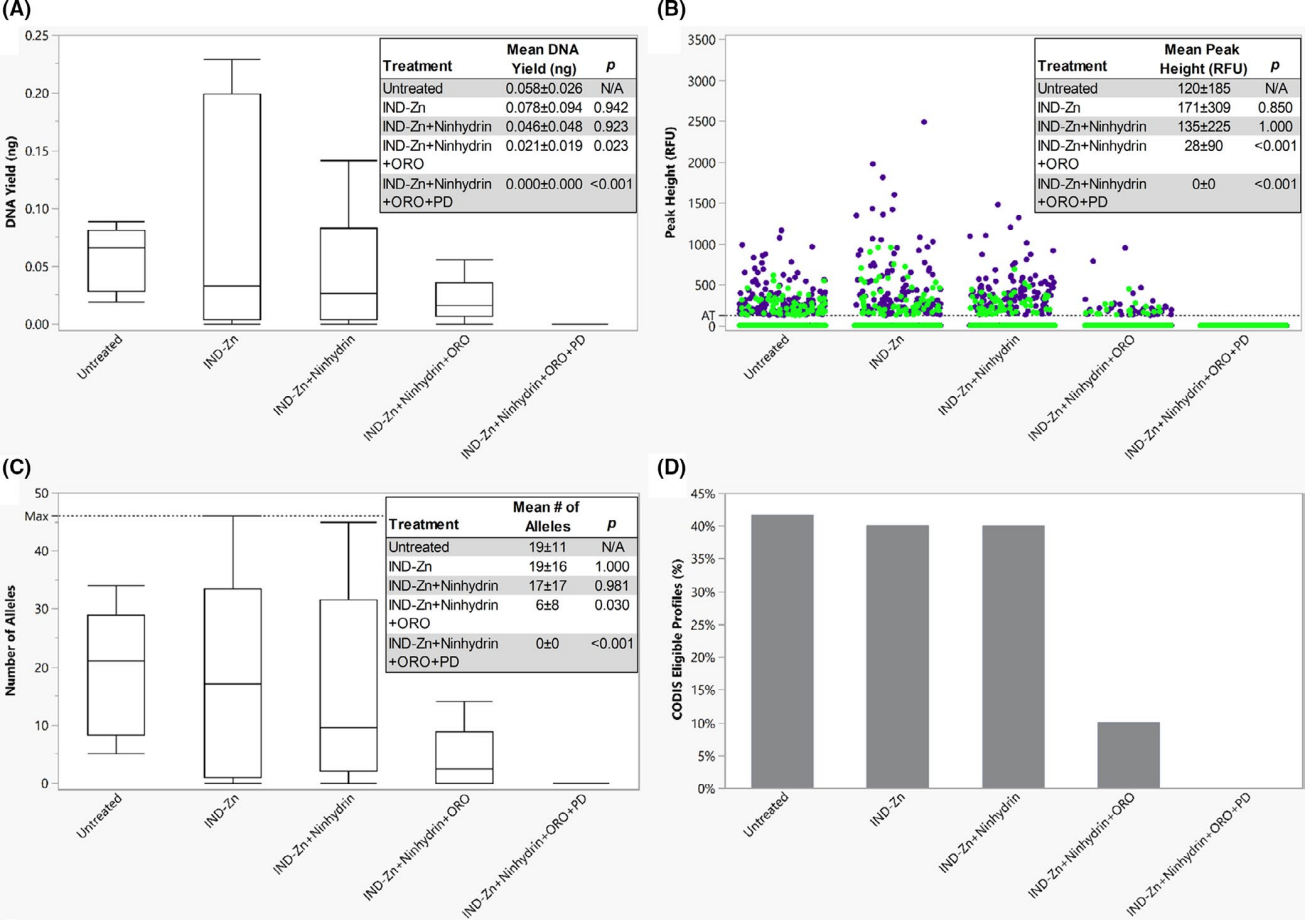
Latent fingerprints developed using sequential processes (A) A boxplot of the DNA yield for each process, showing the median and the 1st and 3rd quartiles. The whiskers represent 1.5 times the interquartile range. (B) The distribution of peak heights for lower (<200 bp, blue dot) and higher (>200 bp, green dot) molecular weight loci, with an AT of 125 RFU. (C) A boxplot of the number of alleles obtained following each process, showing the median and the 1st and 3rd quartiles. The whiskers represent 1.5 times the interquartile range. (D) The percentage of profiles eligible for CODIS upload (≥8 loci with a match rarity of at least 1 in 10 million) for each process. All overlaid boxes indicate means ± standard deviations and *p*‐values obtained from comparisons to the untreated samples (Steel‐Dwass All Pairs, *α* = 0.05) [Color figure can be viewed at wileyonlinelibrary.com]

**FIGURE 5:**
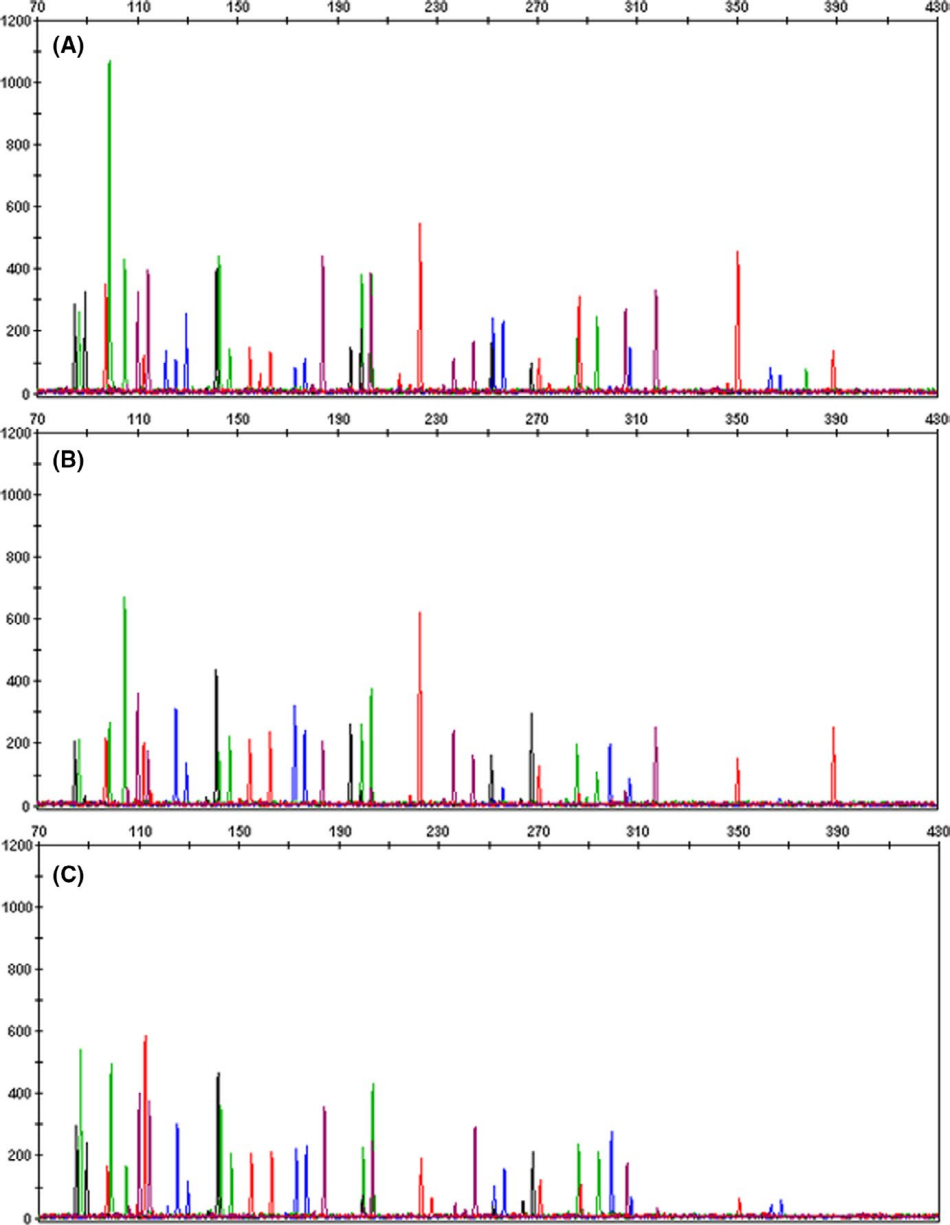
(A) An untreated fingerprint generated a partial profile with 34 alleles. The DNA yield was 0.065 ng, and the average peak height was 228 ± 204 RFU. (B) A fingerprint treated with IND‐Zn generated a partial profile with 33 alleles. The DNA yield was 0.055 ng, and the average peak height was 182 ± 144 RFU. (C) A fingerprint treated with IND‐Zn + ninhydrin generated a partial profile with 29 alleles. The DNA yield was 0.069 ng, and the average peak height was 185 ± 171 RFU [Color figure can be viewed at wileyonlinelibrary.com]

Finally, the latent fingerprints treated sequentially with IND‐Zn, ninhydrin, ORO, and PD were compared with the untreated latent fingerprints (Figure 4). Significantly lower DNA yields were obtained from the samples treated with IND‐Zn + ninhydrin + ORO (*p* = 0.023) and IND‐Zn + ninhydrin + ORO + PD (*p* < 0.001) when compared with the untreated samples. Samples treated with IND‐Zn + ninhydrin + ORO + PD also produced significantly lower DNA yields than IND‐Zn, IND‐Zn + ninhydrin, and IND‐Zn + ninhydrin + ORO (*p* = 0.007). Mean peak heights decreased after the addition of each treatment (IND‐Zn, 171 ± 309 RFU; IND‐Zn + ninhydrin, 135 ± 225 RFU; IND‐Zn + ninhydrin + ORO, 28 ± 90 RFU; and IND‐Zn + ninhydrin + ORO + PD, 0 ± 0 RFU), although the differences between IND‐Zn and IND‐Zn + ninhydrin were not significant (Figure 5). Significantly lower peak height values were obtained following IND‐Zn + ninhydrin + ORO processing when compared with the untreated samples and IND‐Zn‐treated samples (*p* < 0.001). Development with PD after ORO resulted in no peaks above the AT, and comparisons to the untreated samples and the previous treatments were statistically significant (*p* < 0.001). Following this trend, the number of alleles obtained decreased after each successive treatment. Significantly fewer alleles were observed in samples treated with IND‐Zn + ninhydrin + ORO (*p* = 0.030) and IND‐Zn + ninhydrin + ORO + PD (*p* < 0.001) when compared with the untreated samples. IND‐Zn + ninhydrin + ORO + PD treatment also produced significantly fewer alleles than IND‐Zn (*p* = 0.002), IND‐Zn + ninhydrin (*p* = 0.002), and IND‐Zn + ninhydrin + ORO (*p* = 0.019). Despite decreases in DNA yield and number of alleles obtained compared with the untreated samples, 40% of profiles resulting from samples treated with IND‐Zn and IND‐Zn + ninhydrin met the threshold for CODIS eligibility. After treatment with ORO, the number of CODIS eligible profiles dropped to 10%, and no samples treated with PD produced CODIS eligible profiles.

## 4 DISCUSSION

Latent fingerprints on copy paper treated with single‐reagent and sequential fingerprint development processes produced DNA profiles with varying degrees of success. The single‐reagent treatments that were the least harmful to downstream DNA analysis were IND‐Zn and IND‐Zn/laser, a conclusion that has been demonstrated previously [33]. For the sequential treatments containing IND‐Zn and IND‐Zn/laser, the DNA yield and overall quality of the STR profiles generally decreased with the addition of each sequential treatment (e.g., ninhydrin, ORO, PD). As more treatments are used, the opportunities for DNA loss or damage increase. For example, DNA loss occurs when cells are washed away during sample immersion, which will recur with repeated immersions. In low‐yield samples, such as fingerprints, any DNA loss is particularly detrimental to the resultant STR profiles and may result in increased allelic dropout or stochastic effects.

This study did not determine whether decreases in DNA yield and STR profile quality were due to DNA loss resulting from immersion of the samples during latent fingerprint development; DNA degradation resulting from exposure to high heat, high humidity, incompatible pH, or deleterious chemicals; or a combination of these factors. Heat induces DNA degradation through a variety of mechanisms that can lead to strand breakage [34], including hydrolysis [35, 36], deamination [37, 38], depurination [39], depyrimidination [40], and oxidation [41, 42, 43]. Degradation rates resulting from these mechanisms increase as temperature and incubation times increase and pH decreases [44]. Various temperatures and incubation times have been used to promote damage to DNA both in aqueous solution [45, 46, 47, 48] and under dry conditions [49, 50]. Brisco et al. [46] found that DNA heated to 99°C for 20 min demonstrated a ten‐fold increase in lesions/1000 bases when compared with unheated DNA. Salata et al. [47] found minimal degradation following qPCR of 007 control DNA that was incubated at 99°C for 1 h. Machida et al. [48] incubated K562 DNA at 95°C for 5 min to 6 h and demonstrated progressive DNA fragmentation; full 14‐locus STR profiles were obtained from samples incubated for ≤15 min, after which high partial to no profiles were obtained depending on the length of incubation. No hard guidelines exist regarding the impact of various temperatures and incubation times on subsequent forensic DNA processing. Generally, 100°C is considered the upper limit for performing DNA denaturation, although the impact of temperature on the DNA will be affected by other variables, including incubation time, humidity, pH, pressure [39], salt concentration, hydrolysis medium [45], and DNA concentration [45]. High temperatures (90–100°C), high pH (pH >11.3), and some organic solvents (e.g., methanol) [51, 52, 53] also cause DNA denaturation. Depurination and deamination of single‐stranded DNA occur at increased rates when compared with double‐stranded DNA [39, 44, 54].

The most detrimental treatments were DFO, DFO/laser, and PD. Single‐reagent and sequential treatments containing DFO, DFO/laser, or PD were not successful and exhibited statistically significant decreases in DNA yield, peak height, and number of alleles obtained when compared with the untreated samples. DNA loss and poor quality STR profiles following DFO and DFO/laser treatments may be attributed to incubation of the samples at 100°C for 20 min in a dry oven. In addition to longer exposure to higher temperatures, the degradative effect may be exacerbated if the DNA is subjected to methanol and higher concentrations of acetic acid during processing with the DFO solution. Treatment with PD has previously been found to be detrimental to DNA processing, most likely due to the extremely low pH of the maleic acid and PD working solutions (pH ~ 1) [1]. Additional issues may arise from the presence of metal ions in the PD working solution, which enhance the formation of reactive oxygen species and encourage oxidative DNA damage through the Fenton reaction [42, 43]. When planning to perform downstream DNA processing of latent fingerprints, these treatments should be avoided both in both single‐reagent and sequential processes.

To determine the effect of the laser treatment on DNA recovery, DNA was collected from DFO and IND‐Zn‐treated fingerprints with and without exposure to the laser. Although DNA profiling was not particularly successful after either DFO or DFO/laser treatment, the results indicated that use of the laser did not significantly impact the number of alleles obtained. Despite this, some consideration should be given before using the laser in conjunction with IND‐Zn as the percentage of profiles eligible for CODIS upload decreased from 40% to 20% after the laser was used.

Despite using one donor to remove inter‐donor variations related to individuals' differing propensities to shed epithelial cells, intra‐donor variability was observed when examining the untreated control fingerprints. DNA was recovered from all untreated fingerprints (0.018–0.089 ng); however, only 42% yielded CODIS‐eligible STR profiles, and the number of alleles per profile varied widely (5–34 alleles). Previous studies have shown that replication of DNA results from fingerprint samples deposited by one donor can be challenging due to intra‐donor variation caused by a number of factors [55, 56, 57, 58, 59, 60]. During sample preparation, factors that contribute to intra‐donor variation were mitigated as much as possible by providing the donor with guidelines regarding hand washing and the duration and amount of pressure to be used when depositing the fingerprints; however, the amount of DNA contained within a fingerprint cannot be fully controlled or standardized.

Optimization of the DNA collection and processing methods used after latent fingerprint development may result in improved DNA recovery and better quality STR profiles. Wet swabbing with cotton swabs was the only collection method examined in this study; however, other methods, such as cutting samples from the paper, may prove more effective. Cotton swabs can retain up to 50% of the recoverable DNA [61]. Furthermore, DNA extraction can result in the loss of ≥72% of the initial template amount [62, 63], and alternative processing methods may improve the overall quality of the STR profiles. In particular, direct amplification, a method in which a cutting or swab is added directly to an amplification reaction without prior extraction or quantification, has been identified as an effective method for improving DNA profiles from low‐yield samples [64].

## 5 CONCLUSIONS

Single‐reagent and sequential fingerprint development treatments can be used to visualize latent fingerprints on paper items for examination by fingerprint examiners and for targeted DNA collection by DNA analysts. While latent fingerprint enhancement can help provide more information during an investigation, the number and types of fingerprint development treatments that are used can negatively impact the ability to obtain DNA from the fingerprints. In particular, the use of single‐reagent and sequential latent fingerprint development treatments containing DFO and PD are not recommended when performing downstream DNA analysis as these treatments have been found to be detrimental to DNA processing. Prior to examination, fingerprint examiners and DNA analysts should determine which forensic analyses will be performed to facilitate the selection of a single‐reagent or sequential fingerprint development treatment that maximizes fingerprint visualization and minimizes interference with the development of CODIS eligible DNA profiles. Although selection of appropriate development treatments can minimize the opportunities for DNA loss and damage, the development of CODIS‐eligible DNA profiles is not guaranteed due to the variable amounts of DNA contained within fingerprints.
